# Long-term survival of resurfacing humeral hemiarthroplasty

**DOI:** 10.1007/s00590-024-04010-9

**Published:** 2024-05-29

**Authors:** Simo S. A. Miettinen, Yang Liu, Heikki Kröger

**Affiliations:** 1 Department of Orthopaedics, Traumatology and Hand Surgery, Kuopio University Hospital, P.O. Box 1777, 70211 Kuopio, Finland; 2 Kuopio Musculoskeletal Research Unit (KMRU), Faculty of Health Sciences, University of Eastern Finland, Yliopistonranta 1, 70210 Kuopio, Finland

**Keywords:** Shoulder replacement, Shoulder hemiarthroplasty, Arthroplasty, Shoulder, Survival, Complications

## Abstract

**Introduction:**

The indication for shoulder resurfacing arthroplasty is controversial, and survival of these implants is somewhat inconsistent. This study aimed to evaluate the long-term survivorship of resurfacing humeral head implants (RHHIs) and determine risk factors for complications and revisions.

**Materials and methods:**

This retrospective cohort study consisted of 275 cases and two types of RHHIs. The survival rate was evaluated using the Kaplan–Meier method and Cox regression for risk factor analysis. Demographic factors were studied, and the change in the humerus centre of rotation (COR) was measured.

**Results:**

The mean follow-up time was 8.7 years (SD 2.7 months, range 2.8–15.9 years). The mean age of the patients was 67.6 years (SD 9.6, range 33.5–84.9). Ten-year cumulative RHHI survival was 94.1%. The cumulative estimate without any complication was 98.9% at 5 years, 80.0% at 10 years and at 15 years it was 61.5%. The most common complication was persistent pain in 13.8% of the RHHIs. The risk factors for complications and revisions were implant type, preoperative conditions and change of COR > 5 mm.

**Conclusions:**

RRHIs showed excellent long-term survival, but many complications were found. The most common complication was persistent pain, which seemed to be caused by a change of COR and was more related to one type of implant.

## Introduction

Shoulder arthroplasty is performed on a shoulder joint where cartilage destruction following osteoarthritis (OA), avascular necrosis (ANV), rheumatoid arthritis (RA) or post-traumatic injury has progressed to an advanced stage, causing pain and limitation of movement [1]. There are four types of implant systems for shoulder arthroplasty. A resurfacing humeral head implant (RHHI) is designed to replace the damaged joint surfaces and restore normal anatomy with minimal resection of bone [1]. In the 1990s and 2000s, the RHHI gained popularity as studies showed good functional results with low complication and revision rates [2–9]. Currently, the most popular shoulder implant systems are total shoulder arthroplasty (TSA) and reverse shoulder arthroplasty (RSA) [1, 5, 8]. The fourth type of implant is hemiarthroplasty (HA), which is mainly used to treat a fragmented proximal humerus fracture [10].

Treating shoulder arthritis in young active patients remains challenging [2, 9, 11–13]. The experience of shoulder arthroplasty with stemmed implants in young patients has shown worse results than in older patients, with a high percentage of unsatisfactory results and revision surgeries [8, 9, 11–13]. The superiority of TSA in terms of return to sports, function and patient satisfaction over RHHI and HA has been demonstrated in patients with glenohumeral arthritis. Still, some surgeons may prefer them in high-demand athletes and labourers [13]. The most common causes of RHHI revision are inflammation, periprosthetic fractures, luxation and instability, component detachment and damage to the rotator cuff [2, 5, 6, 8, 9, 12]. RHHI revision is often a demanding and costly procedure, with the outcome often somewhat unsatisfactory, especially in young patients [8, 9]. As RHHI differs in many aspects from stemmed shoulder prosthesis, it has some advantages, which are preserved bone stock as bone resection is minimal, short operation time, less stress shielding, a low prevalence of humeral periprosthetic fractures, ease of stem removal at revision and humeral head placement independent from the anatomic axis [7–9]. In addition, RHHIs have been associated with low complication rates [3–5, 7–9]. Moreover, if the RHHI fails, revision surgery can be easily achieved by a revision to a stemless reverse prosthesis, as the bone stock has been maintained, no cement or stem must be exposed and removed, and no loss of length will be encountered [8, 10]. However, bone stock alterations are common after TSA and complicate revision surgery, which may affect the revision threshold level.

The purpose of the present study was (1) to analyse RHHI survivorship at a minimum follow-up of 5 years, (2) to determine complications and revision rates and (3) to evaluate risk factors for complications and revisions.

## Materials and methods

### Setting, participants and implants

This was a retrospective cohort study of consecutive cases performed at a single institution. This study consisted of patients who received primary RHHIs between 1 January 2005 and 31 December 2015. A minimum of 5 years of follow-up was set. The end of follow-up was either 31 December 2020, upon the patient’s death or the date of revision due to any reason. A total of 275 cases (257 patients; 18 bilateral procedures) were evaluated, and 158 of 275 cases (58%) were women. None of the hospital’s RHHI patients were excluded from this study.

Data were retrieved from the hospital medical records, including demographic data [gender, age, operating side, indication for operation and past medical conditions or medications that may affect prognosis (e.g. rheumatism, osteoporosis/-penia, long-term cortisone use, heavy alcohol use, smoking), body mass index (BMI)]. Shoulder post-operative range of motion in flexion and abduction were given in medical records at the 3-month follow-up. However, unfortunately, in most cases, preoperative range of motion and especially post-operative internal and external rotation were reported incompletely, so they were not evaluated. Complications and revisions were evaluated.

### Implant description, surgical technique and follow-up

**Fig. 1:**
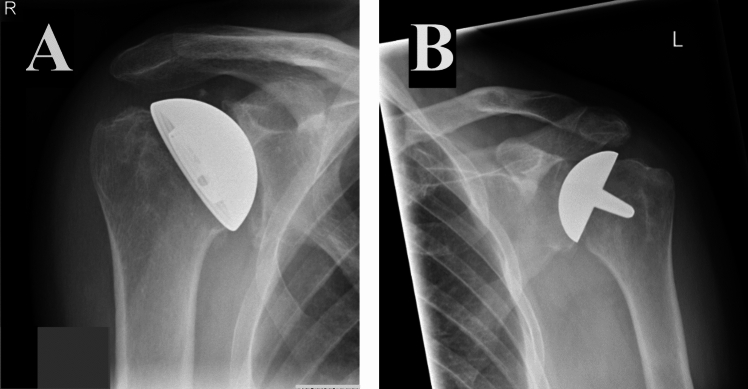
Postoperative radiographs of the Epoca **A** and Copeland **B** humeral head resurfacing arthroplasties

Two types of cementless resurfacing implants were studied (Fig. 1). Epoca (Synthes, Paoli, Pennsylvania, USA) is a Neer-3 type anatomical humeral head resurfacing implant. Copeland (Biomet Inc., Warsaw, Indiana, USA) is a resurfacing implant with porous hydroxyapatite coating for cementless fixation.

Preoperative evaluation included anteroposterior radiographs. Four experienced consultant orthopaedic surgeons performed these operations during the study period. The resurfacing procedure was performed with both general and regional anaesthesia. The patient was placed in a supine, beach-chair position, and the deltopectoral approach was used. The rotator cuff was assessed, and if it was intact or deficiency was repairable (partial small tear), an RHHI was performed. The rotator interval was identified, and the subscapular tendon was incised longitudinally and detached. The humeral head was dislocated anteriorly. The anatomical neck of the humerus was defined, and the neck-shaft angle was identified. All osteophytes were removed, and the humeral shaper was applied to the centre of the head. The humeral drill guide was positioned in the centre of the head and parallel to the neck, adjusting automatically for retroversion and inclination. The humeral head was measured and then reamed over the drill guide. The trial humeral cap was set, and the range of movement was confirmed. If the trial component was correct in size, it was removed, and the final implant was hammered to a prepared humeral head. The subscapularis was re-attached to its insertion, and if a partial rotator cuff tear was noticed, it was repaired with transosseous sutures.

After the RHHI, patients followed a routine rehabilitation protocol with supervised physiotherapy. Patients were routinely followed clinically and radiologically 12 weeks after the procedure and, if needed, 6 or 12 months after the procedure. If problems occurred, extra clinical and radiological follow-up was set, e.g. in case of persistent pain.

### Radiographic measurements

**Fig. 2:**
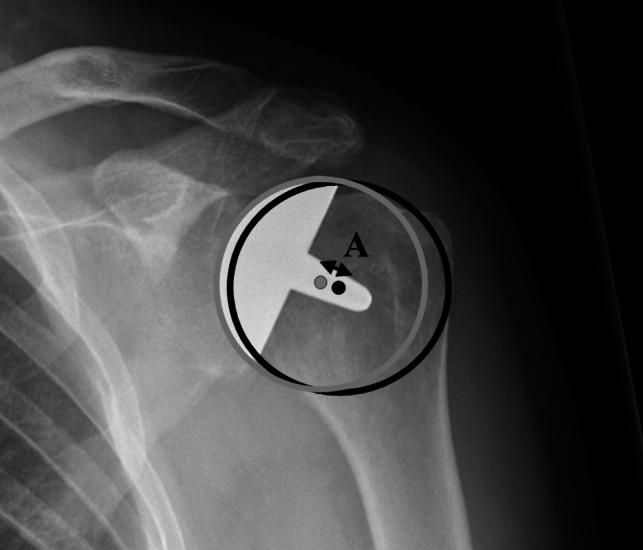
Measurement of the difference of the centre of rotation **A**, where the “true” centre of rotation dot is coloured black and implant-matched dot is coloured grey

Anteroposterior (AP) radiograph measurements were performed to estimate the correct size and position of the implant. For this evaluation, the centre of rotation (COR) was measured by placing a circle on the "true" AP radiograph using three preserved bone landmarks: The lateral cortex of the greater tubercle, medial calcar at the inflection point where calcar meets the articular surface, and the medial edge of the greater tubercle medial of the footprint of the supraspinatus tendon. A second circle, the implant-matched circle, was placed to fit the curvature of the prosthetic humeral head. The COR was identified from each circle, and the distance between the CORs was calculated in millimetres (Fig. 2) [14, 15]. Previously, it has been shown that a COR change greater than 5 mm correlates to clinical failure due to overstuffing and that the COR is a reliable radiologic measurement [14, 15].

### Outcome measures

The outcomes of this study were (1) to analyse RHHI survivorship, (2) to determine complications and revision rates and (3) to evaluate risk factors for complications and revisions.

### Statistical analysis

Kaplan–Meier and log-rank tests were used to study post-operative cumulative survival, and competing risk analysis was performed where death was set as a competing outcome factor for revision. Continuous variables are presented as means (ranges), whereas categorical variables are presented as absolute frequencies and percentages. Comparison of continuous data was carried out using a Mann–Whitney U test. For categorical data, a Chi-square test was used. Cox regression analysis was used to evaluate the most common risk factors for revision (age, age > 65 years, gender, implant, COR, disease affecting bone strength, BMI). A *p*-value < 0.05 was considered statistically significant. The data were analysed using Statistical Package for the Social Sciences (SPSS Inc., Chicago, IL, USA. Ver 27.0, IBM).

## Results

### Patients and demographics

The mean follow-up time was 8.7 years [standard deviation (SD) 2.7 months, range 2.8–15.9 years]. The mean age of the patients was 67.6 years (SD 9.6, range 33.5–84.9). The mean BMI was 29.1 (SD 5.6, range 17.7–54.9). Demographic preoperative characteristics of the patients are given in more detail in Table 1. The mean 3-month post-operative range of abduction angle was 109° (SD 33.5, range 30–180), and the mean flexion angle was 116° (SD 33.3, range 40–180). The mean COR was 3.6 mm (SD 3.0, range 0.0–17.0). A change in the COR > 5 mm was found in 67 of 275 (24%) RHHIs.

**Table 1:** Patient characteristics

	n (%)
Implant*	
Copeland	145 (53)
Epoca	130 (47)
Age > 65 years	
Yes	161 (59)
No	114 (41)
Operation side	
Right	149 (54)
Left	126 (46)
Operation diagnosis	
Primary osteoarthritis	242 (88)
Post-traumatic osteoarthritis	22 (8)
Rheumatoid arthritis	11 (4)
Conditions affecting bone strength and ambulatory status	
None	223 (81)
Osteopenia/-porosis	17 (6)
Rheumatoid arthritis	31 (11)
Heavy use of alcohol	1 (0)
Smoking	3 (1)

*The Mann–Whitney U test and a Chi-square test were applied to compare demographic factors between the implants. None of the statistical tests reached significance

### Complications and revisions

**Fig. 3:**
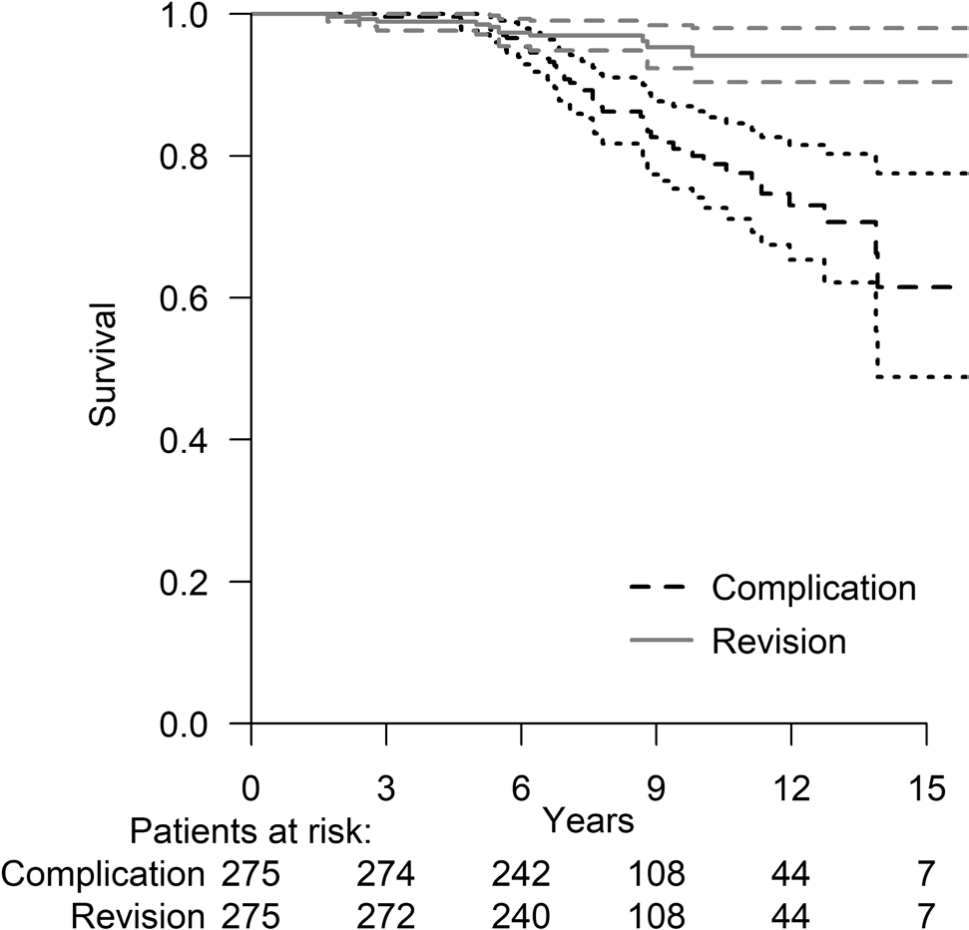
Kaplan–Meier survival analysis for time. Competing risk analysis was performed where death was set as a competing outcome factor for revision. The cumulative estimate without any complication (black dotted line) after RHHI was 98.9% at 5 years, 80.0% at 10 years and at 15 years it was 61.5% (SE 0.3, CI 95% 13.0–14.2). The cumulative estimate without revision (grey solid line) after RHHI was 98.5% at 5 years and 94.1% at 10 years and at 15 years (SE 0.2, CI 95% 15.1–15.7)

A total of 46 of 275 (16.7%) complications occurred, and a total of 11 of 275 (4.0%) revisions were performed (Table 2). The cumulative estimate without any complication after the RHHI was 98.9% at 5 years, 80.0% at 10 years and at 15 years it was 61.5% (SE 0.3, CI 95% 13.0–14.2) (Fig. 3). Of the RHHIs, 52 of 257 (20%) patients died during follow-up at different time points, which was considered in competing risk survival analyses. The cumulative estimate without revision after an RHHI was 98.5% at 5 years and 94.1% at 10 and 15 years (SE 0.2, CI 95% 15.1–15.7) (Fig. 3). In all revision cases, revision was performed with RSA.

**Table 2:** Complications and revisions

	n (%)
Any complication	
No	229 (83.3)
Yes	46 (16.7)
Persistent pain	38 (13.8)
Periprosthetic fracture	6 (2.2)
Implant loosening	1 (0.3)
Nerve damage	1 (0.3)
Revision	
No	264 (96.0)
Yes	11 (4.0)
Persistent pain	8 (2.9)
Periprosthetic fracture	2 (0.7)
Implant loosening	1 (0.4)

### Risk factor analysis

The Cox regression analysis (Table 3) showed that the Copeland implant was a risk factor for complications and revision. If the patient had any conditions affecting bone strength and ambulatory status, it was a risk factor for complications but not for revision. A change of the COR > 5 mm was a risk factor for complications and revision.

**Table 3:** Cox regression analysis for complication and revision risk factors

	Complication						Revision					
	Univariate			Multivariate			Univariate			Multivariate		
	HR	95% CI	*p*-value	HR	95% CI	*p*-value	HR	95% CI	*p*-value	HR	95% CI	*p*-value
Age	1.0	0.98–1.03	0.780				0.98	0.92–1.04	0.450			
< 75 years	ref						ref					
≥ 75 years	1.14	0.63–2.03	0.670				0.86	0.27–2.80	0.800			
Gender												
Female	ref						ref					
Male		0.63–2.02	0.700				1.77	0.51–6.12	0.370			
Implant												
Epoca	ref						ref					
Copeland	0.42	0.23–0.78	0.006	0.47	0.25–0.91	0.024	0.15	0.03–0.77	0.023	0.17	0.03–0.93	0.040
Operation indication†												
Osteoarthritis	ref											
Post-traumatic arthritis	0.37	0.08–1.61	0.180									
Rheumatoides arthritis	0.39	0.12–1.32	0.130									
BMI	1.03	0.98–1.08	0.300				1.06	0.98–1.15	0.130			
Conditions affecting bone strength and ambulatory status*	0.49	0.25–0.94	0.033	0.51	0.27–0.99	0.046	0.56	0.15–2.03	0.380			
Change of centre of rotation > 5 mm	0.91	0.83–1.00	0.046	0.94	0.85–1.03	0.200	0.84	0.73–0.98	0.023	0.90	0.76–1.06	0.200

*CI* Confidence interval, *BMI* Body mass index

†Only 1 revision in other group than primary osteoarthritis so Cox regression was not able use for revision risk factor analysis

*None of the single condition reached statistical significance

## Discussion

The present study showed good long-term results with a cumulative survival of 94.1% at 10 and 15 years after cementless RHHI. In the literature, the results of humeral surface replacement arthroplasty and other shoulder arthroplasties are inconsistent due to different study protocols, operation indications, implants, threshold levels for revision and endpoints. The recent Cochrane review included 20 randomised studies of any type of shoulder replacement, and the conclusion was that it remains uncertain which type or technique of shoulder replacement surgery is most effective in different situations [16]. In a previous study with similar cementless RHHI implants, short-term survival was much worse than in our study, as Maier et al. reported their revision rate as high as 24% after 2.7 years [17]. In contrast to those results, Levy et al. reported no revision after 4.4 years of follow-up, and Al-Hadithy et al. reported a revision rate of 2% after 4.2 years [3, 18]. In the mid-term follow-up studies, reported revision rates varied from 16 to 17% [19, 20]. In the long-term follow-up studies, Geervliet et al. reported a revision rate of 23% at 9 years, and Gadea et al. reported a 10-year prosthesis survival of 88.1% [15, 21]. In our study, there would have been more revisions if RSA had been an option during the early years of the study period. The change in the number of revisions in this study was seen during the latter study period when RSA became available in our clinic.

Many complications (16.7%) occurred in this study, most of which were persistent pain. Similar results have been shown in a recent long-term study where the overall complication rate was 15.4%, and the two main complications were pain due to glenoid erosion and stiffness [21]. The two reasons for the pain were related to RHHI positioning and overstuffing [17, 20, 22]. Moreover, pain after an RHHI has been a problem, especially with the Copeland implant, as overstuffing seems to cause pain due to the implant design [22]. In our study, the Copeland implant was also a risk factor for complications and revision. However, the number of revisions in our study was too small for sound implant-based comparison.

A change in the COR from its anatomical location causes problems. Positioning of the RHHI related to the top of the greater tuberosity is fundamental to avoid impingement of the greater tuberosity under the acromion if it is placed too low or overstuffing the cuff tendons with limited range of motion if it is placed too proud [17]. Our study agrees that increased COR is a risk factor for revision by causing joint pain and stiffness typically referred to as glenoid erosion, impingement or overstuffing [17, 19, 21].

The indication for RHHI has been shown to affect the risk of complications and survival [21]. In the case of primary OA, studies have shown that TSA produces better outcomes for pain, mobility and revision rates compared to RHHI [23, 24]. In the case of RA-indicated RHHI, a study by Sterling et al. showed that long-term pain relief was better after TSA than after hemiarthroplasty, and TSA had a lower risk for revision surgery [25]. In our study, the indication for RHHI was not a risk factor for revision. Implant survival is crucial, especially in younger patients, and a previous study showed that TSA is superior compared to RHHI in patients under 50 years [11]. In this study, the patient’s age was not a risk factor for revision. However, conditions affecting bone strength and ambulatory status are risk factors for complications but not for revision. The probable reason for this finding is that patients with complications might have poor glenoid bone quality, causing glenoid erosion, and the rotator cuff might be degenerative. However, as these patients were not suitable for RSA revision for multiple reasons, these are risk factors only for complications and not for revision in this current study. Gender or BMI were not risk factors for complications or revision in this study.

The current study has several limitations. Due to the retrospective study design, no patient-reported outcome measurements were available. The options for RHHI revisions were minimal during the early study period due to the lack of TSA and RSA options. This may have resulted in too low a revision rate during the early study period. On the other hand, in the latter study period, when RSA became an option, the patients who would have benefited from revision were in too poor condition for another surgery. There was no radiological analysis in the axial plane, hence limiting the evaluation of the resurfacing. The use of 3-D computed tomography would be more reproducible than plain radiography assessment [26]. In addition, we did not consider the radiographic data at baseline, and glenoid cavity morphology was not analysed.

## Conclusion

RRHIs showed excellent long-term survival, but many complications were found. The most common complication was persistent pain. The lack of good revision options for these patients limited the number of revisions, especially in the 2000s. In the 2010s, these problematic patients were mostly too fragile for RSA revision, which probably explains the excellent implant survival in this study.

## Data Availability

Not available due to data protection reasons.
